# A phase I trial of pan-Bcl-2 antagonist obatoclax administered as a 3-h or a 24-h infusion in combination with carboplatin and etoposide in patients with extensive-stage small cell lung cancer

**DOI:** 10.1038/bjc.2012.21

**Published:** 2012-02-14

**Authors:** A A Chiappori, M T Schreeder, M M Moezi, J J Stephenson, J Blakely, R Salgia, Q S Chu, H J Ross, D S Subramaniam, J Schnyder, M S Berger

**Affiliations:** 1 H. Lee Moffitt Cancer Center and Research Institute, Thoracic Oncology Program, FOB1, Room 5.111, 12902 Magnolia Drive, Tampa, FL 33612, USA; 2 Clearview Cancer Institute, Huntsville, AL 35805, USA; 3 Integrated Community Oncology Network, Jacksonville, FL 32256, USA; 4 Institute for Translational Oncology Research, Greenville, SC 29605, USA; 5 The West Clinic, Memphis, TN 38120, USA; 6 Department of Medicine, Section of Hematology/Oncology, University of Chicago, Chicago, IL 60637, USA; 7 Department of Medical Oncology, Cross Cancer Institute, Edmonton, Alberta, Canada T6G 1Z2; 8 Mayo Clinic Arizona, Scottsdale, AZ 85259, USA; 9 Department of Internal Medicine, Division of Hematology-Oncology, Georgetown University, Washington, DC 20007, USA; 10 Gemin X Pharmaceuticals, Malvern, PA 19355, USA

**Keywords:** small cell lung cancer, apoptosis, Bcl-2 gene family, CNS symptoms

## Abstract

**Background:**

Bcl-2 family genes are frequently amplified in small cell lung cancer (SCLC). A phase I trial was conducted to evaluate the safety of obatoclax, a Bcl-2 family inhibitor, given in combination with standard chemotherapy.

**Methods:**

Eligible patients (3–6 per cohort) had extensive-stage SCLC, measurable disease, ⩽1 before therapy, Eastern Cooperative Oncology Group performance status 0 or 1, and adequate organ function. Patients were treated with escalating doses of obatoclax, either as a 3- or 24-h infusion, on days 1–3 of a 21-day cycle, in combination with carboplatin (area under the curve 5, day 1 only) and etoposide (100 mg m^−2^, days 1–3). The primary endpoint was to determine the maximum tolerated dose of obatoclax.

**Results:**

Twenty-five patients (56% male; median age 66 years) were enrolled in three dose cohorts for each schedule. Maximum tolerated dose was established with the 3-h infusion at 30 mg per day and was not reached with the 24-h infusion. Compared with the 24-h cohorts, the 3-h cohorts had higher incidence of central nervous system (CNS) adverse events (AEs); dose-limiting toxicities were somnolence, euphoria, and disorientation. These CNS AEs were transient, resolving shortly after the end of infusion, and without sequelae. The response rate was 81% in the 3-h and 44% in the 24-h infusion cohorts.

**Conclusion:**

Although associated with a higher incidence of transient CNS AEs than the 24-h infusion, 3-h obatoclax infusion combined with carboplatin–etoposide was generally well tolerated at doses of 30 mg per day. Though patient numbers were small, there was a suggestion of improved efficacy in the 3-h infusion group. Obatoclax 30 mg infused intravenously over 3 h on 3 consecutive days will be utilised in future SCLC studies.

Small cell lung cancers (SCLCs) account for approximately 13% of all lung cancer cases (Govindan *et al*, 2006), and two-thirds of patients with SCLC have extensive stage (ES) disease and are not considered to be curable. Despite initial tumour chemosensitivity, median survival for patients with ES-SCLC is 7 to 10 months, and the 2-year survival rate is <5% (Thatcher *et al*, 2005) due to frequent relapse with metastatic and/or chemoresistant disease.

Platinum-based chemotherapy (cisplatin or carboplatin) combined with etoposide is generally recommended as the initial therapy (Spira and Ettinger, 2004; also see NCCN Guidelines at www.nccn.org). Four to six cycles of this combination are considered optimal (Thatcher *et al*, 2005). Because brain metastases are common in patients with ES-SCLC, prophylactic cranial irradiation is generally recommended in patients with ES-SCLC who achieve a response to systemic chemotherapy to prolong median overall survival (Slotman *et al*, 2007).

BCL-2 family proteins are frequently (65–80%) expressed in SCLC (Ben-Ezra *et al*, 1994; Jiang *et al*, 1995), and *Bcl-2* family genes are frequently amplified in SCLC cell lines (Beroukhim *et al*, 2010). Furthermore, SCLC cell lines are sensitive to inhibition of the pro-survival members of the BCL-2 family (Shoemaker *et al*, 2008; Dean *et al*, 2011). Thus, inhibiting the BCL-2 pro-survival proteins may be a useful strategy in the treatment of SCLC.

Obatoclax mesylate (GX15-070MS) is a small molecule that antagonises pro-survival members of the BCL-2 family of proteins by binding the BH3-binding groove and blocking interaction with pro-apoptotic family members. Obatoclax inhibits all BCL-2 pro-survival family members, including MCL-1 (Nguyen *et al*, 2007; Warr and Shore, 2008). It has been shown to activate apoptosis *in vitro* (Nguyen *et al*, 2007) to exhibit antitumour activity in animal models (Nguyen *et al*, 2007; Smoot *et al*, 2010), and to enhance the effects of chemotherapeutic agents, including cisplatin and etoposide, in SCLC cell lines *in vitro* (Dean *et al*, 2011). It has previously shown single-agent biological and clinical activity in phase I studies in other, mainly haematological, malignancies (Schimmer *et al*, 2008; O’Brien *et al*, 2009).

Obatoclax may be associated with infusional central nervous system (CNS) toxicities such as somnolence, euphoric mood, and ataxia (O’Brien *et al*, 2009; Hwang *et al*, 2010). These toxicities may be mediated by inhibition of BCL-XL, a protein that regulates nerve cell synaptic plasticity (Hickman *et al*, 2008). In previous studies, the intensity of infusional CNS toxicities decreased as the infusion duration increased from 1 h to 3 h (Hwang *et al*, 2010), and were even less prominent with longer (24-h) infusions (Schimmer *et al*, 2008).

The safety of the 3-h versus the 24-h infusion schedules has been compared in only one small trial, in older patients, with previously untreated acute myeloid leukemia (Raza *et al*, 2009). Therefore, this study compared the safety and tolerability of the 3-h and the 24-h infusions of obatoclax, combined with carboplatin and etoposide in patients with ES-SCLC.

## Patients and methods

This was an open-label, phase I trial conducted at nine institutions in North America (ClinicalTrials.gov Number NCT00682981). The protocol was approved by the Institutional Review Boards of all participating centres. Informed consent was obtained from all patients before enrolment, in accordance with the Declaration of Helsinki.

### Trial design

The primary objective of this phase I study was to determine the maximum tolerated dose (MTD) and the recommended phase II dose of obatoclax, administered as either a 3-h or a 24-h infusion, when combined with carboplatin and etoposide in patients with ES-SCLC. The secondary objective was to characterise the safety profile of each schedule. Efficacy data for each schedule were also preliminarily assessed.

Three to six patients were enrolled into ascending-dose cohorts. Enrolment alternated between infusion schedules, starting with the lowest 3-h infusion cohort, which was filled before enrolment moved to the lowest 24-h infusion cohort. Planned obatoclax dosage groups were 15, 30, and 45 mg per day with the 3-h infusion, and 30, 45, and 60 mg per day with the 24-h infusion. In each case, the highest dose planned was the single-agent MTD established by previous studies (45 and 60 mg per day, respectively).

Obatoclax was administered first (or, for the 24-h infusion cohort, initiated first), followed by an intravenous infusion of carboplatin (area under the curve 5; day 1 only) and of etoposide (100 mg m^−2^; days 1, 2, and 3), repeated on a 21-day cycle. Obatoclax was administered on days 1–3 to suppress the activity of the BCL-2 family pro-survival proteins during all days of the cytotoxic chemotherapy administration.

Patients received combination cytotoxic chemotherapy treatment for six cycles in the absence of disease progression or intolerable toxicity. Initially, prophylaxis with diphenhydramine and an H2 antagonist was recommended before the administration of obatoclax to prevent cytokine release syndrome. After diphenhydramine was found to exacerbate the infusional somnolence, the protocol was amended to utilise only an H2 antagonist.

All patients who achieved an objective response (complete or partial) with combination chemotherapy were to receive prophylactic cranial irradiation. Patients who remained without progression after chemotherapy were to receive single-agent obatoclax as maintenance therapy every 21 days, using the same dose and schedule administered to that patient during the combination therapy. Maintenance therapy was administered until the occurrence of disease progression or intolerable toxicity. For patients receiving prophylactic cranial irradiation, obatoclax maintenance therapy was not initiated until at least 2 weeks after completion of prophylactic cranial irradiation.

Patients were assessed on the first day of each cycle for the presence of adverse events (AEs). This assessment included a full history and physical examination (emphasising the presence or occurrence of any toxicities), and laboratory blood work, including haematological and biochemical testing. Adverse events occurring during cycle 1 were used to identify dose-limiting toxicities (DLTs) before dose escalation.

For haematological toxicities, DLT was defined as a grade 4 neutropenia with fever, grade 4 neutropenia without fever for ⩾7 days, or grade 4 thrombocytopenia. Grade 3 or 4 non-hematological toxicity not ameliorated by supportive therapy was also defined as a DLT. The MTD was defined as the dose level before the obatoclax dose where two out of six DLTs were observed. Per protocol, the MTD was also considered the recommended phase II dose.

In patients who experienced grade 4 neutropenia, prophylactic growth factor support was recommended in subsequent cycles. For grade 4 neutropenia despite growth factor prophylaxis, or in the case of grade 4 thrombocytopenia, the dose of etoposide was reduced to 80 mg m^−2^, and the dose of carboplatin was reduced to area under the curve 4. For all other grade 3 or 4 AEs unresponsive to supportive care, the dose of obatoclax was reduced by 15 mg per day. Treatment was delayed in patients whose absolute neutrophil count and platelet count had not recovered to ⩾1500/mm^3^ and ⩾100 000/mm^3^, respectively, by the start of the next cycle. Recurrence of toxicity following one dose reduction or a delay in therapy beyond 2 weeks resulted in discontinuation of treatment.

### Patients

Eligible patients were ⩾18 years old, with pathological or cytological confirmation of SCLC and with disease sites consistent with ES-SCLC. Eligibility required measurable disease as defined by the Response Evaluation Criteria in Solid Tumors (RECIST) v1.0 (Therasse *et al*, 2000), as well as the Eastern Cooperative Oncology Group (ECOG) performance status ⩽1 at baseline. Patients with platinum-sensitive relapsed disease were initially allowed. However, the trial was amended to include only chemotherapy-naïve patients after previously treated patients were noted to have a greater degree of myelosuppression. Enrolled patients also had to have normal organ function defined as absolute neutrophil count ⩾1500/mm^3^, platelets ⩾100 000/mm^3^, total bilirubin ⩽upper limit of normal or total bilirubin ⩽3.0 mg dl^−1^ if liver metastases were present, alanine aminotransferase (serum glutamic pyruvic transaminase) ⩽2.5 × upper limit of normal, or alanine aminotransferase (serum glutamic pyruvic transaminase) ⩽5 × upper limit of normal if liver metastases were present, and creatinine within normal limits. A negative pregnancy test result was required for women of childbearing potential, and both women and men had to use a highly effective method of birth control.

Patients with a history of seizure disorders unrelated to SCLC brain metastases or with symptomatic brain metastases were excluded, as were patients who were pregnant or breast feeding.

### Safety and efficacy

Adverse events were graded according to the NCI CTCAE version 3.0. (see http://ctep.cancer.gov/reporting/ctc.html). A computerised tomography or magnetic resonance imaging scan of the brain was obtained at the pre-study visit. Tumour evaluations were documented by either computerised tomography scan with intravenous contrast or magnetic resonance imaging within 21 days of the start of treatment and repeated every other cycle, at the end of treatment visit, and every 6 weeks until progression. Best overall response was assessed using the RECIST v1.0 criteria (Therasse *et al*, 2000). Complete response, partial response, and stable disease were confirmed by a second evaluation at least 4 weeks later.

## Results

### Patients and exposure

Twenty-five patients (median age of 66 years, 56% (*n*=14) male) were enrolled. The ECOG performance status was 0 for 6 patients and 1 for 19 patients. Eighteen of the enrolled patients were chemotherapy-naïve (Table 1). Patients received a median of six combination chemotherapy cycles (range, 2–6), with all six planned treatment cycles completed by 20 patients (80% 16 in the 3-h infusion group, and 4 in the 24-h infusion group). Twelve patients (8 in the 3-h infusion group, and 4 in the 24-h infusion group) went on to receive a median of two (range, 1–24) cycles of maintenance therapy with the single-agent obatoclax. The most common reason for discontinuation was disease progression (*n*=18 (72%)); other reasons for discontinuation included AEs (*n*=3; grade 3 or 4 thrombocytopenia in all three cases), withdrawal by patient (*n*=2), and other (*n*=1). The median duration of treatment was similar for patients on the 3-h infusion schedule (118 days) and patients on the 24-h infusion schedule (115 days). Of the 138 total cycles administered, obatoclax treatment was delayed, interrupted, or discontinued in 22% of cycles (*n*=30) due to AE, and in an additional 9% (*n*=13) due to other reasons; rates of delay/interruption/discontinuation were similar in the 3-h *vs* the 24-h infusion groups. Obatoclax dose reductions were necessary in four patients, all of whom were in the 45 mg per day, 3-h infusion group.

### Dose escalation and MTD

**Figure 1:**
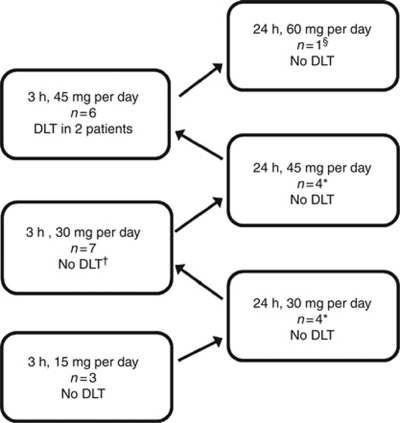
Enrolment into dosing cohorts. A total of 25 patients were enrolled, alternating between 3-h and 24-h infusion cohorts. ^†^No DLT observed in five treatment-naïve patients in this cohort. Myelosuppressive DLT observed in two previously treated patients; protocol was amended to exclude previously treated patients and these DLTs were excluded from dose-escalation decision. ^*^Safety data from one patient excluded from dose-escalation decision due to pump malfunction. ^§^This cohort was discontinued after enrolment of one patient.

Patients were enrolled into six dosing cohorts. The ‘ping-pong’ enrolment alternatively filled cohorts in the 3-h and the 24-h dosing schedules (Figure 1).

In the 3-h infusion arm, no DLTs were observed in the initial cohort treated with obatoclax 15 mg per day. DLT occurred in two patients in the cohort who received obatoclax 30 mg per day over 3 h; in both cases, DLT consisted of myelosuppression in previously treated patients. As a result, the trial was amended to exclude previously treated patients. Five previously untreated patients receiving obatoclax 30 mg per day over 3 h had no DLTs. DLT was observed in two out of six patients who received obatoclax 45 mg over 3 h (somnolence and euphoria, and somnolence and disorientation), establishing the MTD for obatoclax at 30 mg over 3 h for 3 consecutive days (recommended phase II dose).

No DLTs were observed in the patients receiving treatment in the 24-h infusion arm. However, two patients in the obatoclax 24-h infusion cohorts (one at 30 mg per day and another at 45 mg per day) had infusion pump malfunctions while at home. As a result, safety data for these two patients were excluded from the data used for deciding dose escalation. Furthermore, patient safety considerations associated with the potential inaccuracy in the delivery of the obatoclax dose due to pump malfunctions led to early discontinuation of the 60 mg over 24-h dosing cohort. Only one patient was entered in this cohort (this patient had already been enrolled when the decision to discontinue this cohort was made), with no reported DLTs.

### Safety data

The most common AEs reported in the study included neutropenia (96%), thrombocytopenia (76%), anaemia (72%), fatigue (68%), and nausea (52% Table 2). To highlight differences in the safety profile between the 3-h and the 24-h obatoclax infusions, differences in the incidence of non-haematological AEs between the two groups were evaluated (Table 3). CNS AEs (somnolence, euphoric mood, insomnia, and ataxia) occurred at a lower incidence in the 24-h obatoclax infusion group.

Grade 3 and 4 AEs that occurred in two or more patients are displayed in Table 4. Grade 3 or 4 haematological AEs occurring in all patients during the study included neutropenia (75% in 3-h *vs* 89% in 24-h), thrombocytopenia (44 *vs* 33%), anaemia (25 *vs* 22%), and leukopenia (19 *vs* 0%). These data do not indicate large differences in myelosuppression between the obatoclax infusion groups. Non-haematological grade 3 and 4 AEs that occurred with a ⩾10% increase in incidence in either obatoclax infusion group did not exhibit a clear pattern suggestive of toxicity in a particular organ system (Table 3).

Grade 3 or 4 CNS or psychiatric AEs that occurred in this study all occurred in the 3-h obatoclax infusion cohorts. These were grade 3 somnolence in five patients (31% of the 3-h cohort patients), grade 3 syncope in one patient (6%), grade 4 confusional state in one patient (6%), grade 3 depression in one patient (6%), grade 3 euphoric mood in one patient (6%), grade 3 mental status changes in one patient (6%), and grade 3 mood altered in one patient (6%). The majority of these CNS or psychiatric AEs occurred during the obatoclax infusion and resolved shortly after the infusion ended (within the 1-h post-infusion observation period mandated by the protocol). Diphenhydramine appeared to exacerbate the infusional somnolence in some patients. Once this was recognised, the protocol was amended and diphenhydramine was no longer administered prophylactically.

One patient experienced grade 2 cytokine release syndrome. On the first day of cycle 2, after premedication with diphenhydramine and an H2 antagonist, this patient experienced back pain and facial flushing ∼0.5 h after the start of the infusion. The infusion was stopped, and the patient was given additional diphenhydramine. The infusion was re-started, and the patient had no further cytokine release syndrome symptoms through the three additional cycles of the obatoclax treatment that were administered.

Serious AEs were reported in 13 patients (52% 9 in the 3-h infusion group and 4 in the 24-h infusion group) and consisted of anaemia (*n*=2), neutropenia (*n*=2), and nausea (*n*=2), and *n*=1 each of febrile neutropenia, thrombocytopenia, abdominal pain, diarrhoea, oesophagitis, vomiting, chronic obstructive pulmonary disease, hypoxia, pulmonary embolism, labyrinthitis, hyponatremia, syncope, confusional state, and deep vein thrombosis. The incidence of blood and lymphatic system and respiratory disorders reported as serious AEs was similar for the two infusion schedules; GI serious AEs were only reported for patients on the 3-h infusion schedule.

No deaths occurred within 30 days of study treatment. In one patient, treated at the 30 mg per day obatoclax dose level on the 24-h infusion schedule, the AE that led to death was reported as possibly treatment related. This patient received six cycles of treatment before discontinuation due to disease progression; the patient developed grade 2 lung collapse 69 days post-treatment and died approximately 1 month later with the cause of death reported as respiratory failure.

### Clinical efficacy

Table 5 represents the efficacy results for the intent-to-treat population. The overall response rate was 68% (81 *vs* 44% in the 3-h and the 24-h infusion cohorts, respectively). Median progression-free survival in both cohorts was similar, but it was observed that several patients in the 3-h cohorts had considerably longer progression-free survival. The median overall survival was numerically higher in patients who received obatoclax by 3-h infusion (379 *vs* 283 days).

Among chemotherapy-naïve patients, the overall response rate was 83% (15 out of 18), with responses demonstrated in all patients in the 3-h infusion group (*n*=12; 2 complete and 10 partial) and 50% of patients in the 24-h infusion group (3 out of 6; all partial responses). Among the seven patients enrolled who had previously received a platinum-based cytotoxic chemotherapy regimen, three achieved a partial response (one of which was not confirmed). For these three patients, the time between the end of their initial treatment and the start of treatment in this study was 4.5, 6, and 17.7 months. Two were previously treated with carboplatin and etoposide, and one with cisplatin and etoposide. For these seven patients, median progression-free survival was 158 days (76–198) and median overall survival was 364 days (235–504).

## Discussion

In our study, the MTD and recommended phase II dose of obatoclax as a 3-h infusion, in combination with etoposide and carboplatin, were determined to be 30 mg daily for 3 days. As expected, the observed DLTs consisted of CNS AEs previously described with single-agent obatoclax infusions. Somnolence, euphoric mood, and disorientation defined the MTD for the 3-h infusion schedule. The majority of these AEs occurred during obatoclax infusion and resolved shortly after the infusion ended, and did not require any special intervention, except for the standard observation planned in the protocol during and after the drug infusion. There was no MTD defined for the 24-h obatoclax infusion, and only one patient was enrolled at the highest dose level of 60 mg (as the available information was already considered to be adequate to choose between the two schedules).

We observed marked differences in the incidence of somnolence in the 3-h infusion arm (75%) versus the 24-h infusion arm (0%), and in the incidence of euphoric mood in the 3-h infusion arm (44%) versus the 24-h infusion arm (11%). These findings agree with those of previous studies of single-agent obatoclax in which CNS AEs were more frequent and of higher toxicity grade with shorter infusions of obatoclax (Schimmer *et al*, 2008; Hwang *et al*, 2010). Although no direct correlation has been shown between the intensity of CNS symptoms and C_max_ or area under the curve, these symptoms appear to be related to the level of obatoclax exposure. Furthermore, the presence of these symptoms indicates that the drug is able to cross the blood–brain barrier; indeed, the CNS toxicity may be an on-target effect resulting from inhibition of BCL-XL expressed in neurons.

Comparison of the incidence of non-CNS AEs between the two infusion schedules did not indicate major differences or patterns suggestive of specific organ system toxicity. However, increased neutropenia was observed in patients previously treated with chemotherapy. This was likely due to the effects on the bone marrow of prior cytotoxic therapy and not likely due to a direct obatoclax effect, as the overall incidence of neutropenia observed in our trial appears similar to that reported for etoposide and carboplatin in comparable patient populations (Lara *et al*, 2009; Socinski *et al*, 2009).

Navitoclax is another agent in development that inhibits BCL-2 family members BCL-2, BCL-XL, and BCL-W. Phase I evaluation of single-agent navitoclax demonstrated that thrombocytopenia was the dose-limiting AE (Gandhi *et al*, 2011), which is consistent with the role shown for BCL-XL in platelet regulation (Zhang *et al*, 2007). Thrombocytopenia has not been a prominent finding in single-agent phase I trials of obatoclax administered as a 3-h (O’Brien *et al*, 2009) or a 24-h infusion (Schimmer *et al*, 2008). Instead, two patients with myelodysplastic syndrome had marked increases in platelets and became platelet transfusion-independent when treated with single-agent obatoclax (Schimmer *et al*, 2008).

In this trial, grade 3 or 4 thrombocytopenia occurred in 33% of the chemotherapy-naïve patients. This may be a higher rate of grade 3 or 4 thrombocytopenia than the rate of 10% reported in the carboplatin and etoposide control arm of a recent SCLC trial (Socinski *et al*, 2009); however, without a controlled trial, any conclusions in this regard are tentative. Nevertheless, the degree of thrombocytopenia seen with obatoclax in multiple studies appears less than that seen with navitoclax. As plasma exposures of navitoclax in patients treated with this agent (Gandhi *et al*, 2011) are considerably higher than those achieved with obatoclax (O’Brien *et al*, 2009) due to CNS-limiting events, one explanation for the low rate of thrombocytopenia seen with obatoclax may be that obatoclax does not achieve the concentrations in platelets necessary to completely inhibit BCL-XL. Alternatively, it is possible that obatoclax may not inhibit BCL-2 to the same degree as navitoclax.

In this study, it was initially recommended that diphenhydramine and an H2-blocking agent be administered before obatoclax infusion to prevent cytokine release syndrome. Diphenhydramine was recognised to worsen somnolence, and the recommendation was changed to utilise prophylaxis with only an H2-blocking agent. Thus, prophylactic treatment with diphenhydramine is no longer recommended.

Although the study was not powered to compare the efficacy of the two infusion schedules, the overall response rate was higher in patients receiving the 3-h infusion compared with patients receiving the 24-h infusion (81 *vs* 44%), and overall survival was longer in the 3-h infusion cohorts. These data indicate that the 3-h infusion schedule demonstrates promising activity and supports utilising this schedule in future studies.

Data from an earlier study in older patients with previously untreated acute myeloid leukemia also support the conclusion that efficacy with the 3-h infusion of obatoclax may be increased when compared with efficacy with the 24-h infusion (Raza *et al*, 2009). In this study, 13 patients received single-agent obatoclax utilising a 3-h infusion (3 at 20 mg m^−2^ and 10 at 30 mg m^−2^) and 5 received obatoclax utilising a 24-h infusion (all at 60 mg m^−2^), with obatoclax administered on days 1–3 of a 14-day cycle. Efficacy data after cycle 2 indicated that four patients receiving a 3-h infusion of obatoclax had a ⩾50% decrease in bone marrow blasts, compared with none of the patients on the 24-h schedule of obatoclax infusion.

Analysis of pharmacodynamic endpoints in the patients in this trial was performed as a measure of cell death and has been reported elsewhere (Dean *et al*, 2011). These data indicate that patients with radiologic, unconfirmed complete or partial responses after two cycles of treatment (‘responders’) had a significant increase in circulating cell death biomarkers (cleaved cytokeratin 18 and oligonucleosomal DNA) by day 3 with both infusion schedules, whereas nonresponders did not.

There were practical issues seen in the outpatient setting with patients on the 24-h obatoclax infusions that were not seen with the shorter 3-h infusions administered in the infusion suite. Pump malfunctions at home led to the administration of less than full doses for those patients. This difficulty provided an additional reason to favour the 3-h obatoclax infusion schedule in future studies.

Study limitations included the uncontrolled study design, making it difficult to distinguish toxicity related to obatoclax from that related to chemotherapy, and the small number of patients enrolled. Furthermore, pharmacokinetic analyses were not performed in this trial, so precise exposure levels at the different dosages are not available.

Obatoclax administered as a 3-h infusion in combination with carboplatin and etoposide was generally well tolerated at doses of up to 30 mg per day. Although the 3-h infusion was associated with a higher incidence of some (particularly CNS) AEs than the 24-h infusion, preliminary data indicate it may also be associated with improved efficacy compared with obatoclax as a 24-h infusion. In consideration of these preliminary efficacy findings, as well as practical issues with the 24-h infusion arm, 3-h infusions of obatoclax with carboplatin and etoposide are recommended for use in future clinical trials in patients with SCLC.

## Figures and Tables

**Table 1:** Demographic data

	**3-h infusion**			**24-h infusion**			**Total**
Obatoclax dose (mg per day)	15	30	45	30	45	60	—
Number of patients	3	7	6	4	4	1	25
Median age, years (range)	72 (67–75)	65 (53–70)	64 (57–74)	65 (61–69)	70 (65–75)	53 (53)	66 (53–75)
Males	1	3	4	4	2	0	14
							
*ECOG PS*							
0	2	1	2	1	0	0	6
1	1	6	4	3	4	1	19
							
Chemotherapy-naïve	1	5	6	3	2	1	18

Abbreviation: ECOG PS=Eastern Cooperative Oncology Group performance status.

**Table 2:** Most common adverse events (⩾25%) at all toxicity grades

	**3-h infusion schedule, *n* (%)**				**24-h infusion schedule, *n* (%)**				**Total, *n* (%)**
**Preferred term**	**Obatoclax 15 mg per day (*n*=3)**	**Obatoclax 30 mg per day (*n*=7)**	**Obatoclax 45 mg per day (*n*=6)**	**Overall (*n*=16)**	**Obatoclax 30 mg per day (*n*=4)**	**Obatoclax 45 mg per day (*n*=4)**	**Obatoclax 60 mg per day (*n*=1)**	**Overall (*n*=9)**	**(*N*=25)**
Neutropenia	3 (100.0)	6 (85.7)	6 (100.0)	15 (93.8)	4 (100.0)	4 (100.0)	1 (100.0)	9 (100.0)	24 (96.0)
Thrombocytopenia	3 (100.0)	6 (85.7)	4 (66.7)	13 (81.3)	1 (25.0)	4 (100.0)	1 (100.0)	6 (66.7)	19 (76.0)
Anaemia	3 (100.0)	5 (71.4)	4 (66.7)	12 (75.0)	1 (25.0)	4 (100.0)	1 (100.0)	6 (66.7)	18 (72.0)
Fatigue	2 (66.7)	4 (57.1)	6 (100.0)	12 (75.0)	1 (25.0)	3 (75.0)	1 (100.0)	5 (55.6)	17 (68.0)
Nausea	2 (66.7)	3 (42.9)	4 (66.7)	9 (56.3)	2 (50.0)	1 (25.0)	1 (100.0)	4 (44.4)	13 (52.0)
Alopecia	0	5 (71.4)	4 (66.7)	9 (56.3)	1 (25.0)	1 (25.0)	1 (100.0)	3 (33.3)	12 (48.0)
Constipation	3 (100.0)	3 (42.9)	3 (50.0)	9 (56.3)	1 (25.0)	1 (25.0)	1 (100.0)	3 (33.3)	12 (48.0)
Somnolence	1 (33.3)	7 (100.0)	4 (66.7)	12 (75.0)	0	0	0	0	12 (48.0)
Anorexia	2 (66.7)	3 (42.9)	2 (33.3)	7 (43.8)	1 (25.0)	1 (25.0)	1 (100.0)	3 (33.3)	10 (40.0)
Dizziness	2 (66.7)	2 (28.6)	1 (16.7)	5 (31.3)	1 (25.0)	2 (50.0)	1 (100.0)	4 (44.4)	9 (36.0)
Dehydration	2 (66.7)	2 (28.6)	2 (33.3)	6 (37.5)	0	2 (50.0)	0	2 (22.2)	8 (32.0)
Euphoric mood	0	4 (57.1)	3 (50.0)	7 (43.8)	0	1 (25.0)	0	1 (11.1)	8 (32.0)
Hypomagnesemia	2 (66.7)	1 (14.3)	2 (33.3)	5 (31.3)	0	2 (50.0)	1 (100.0)	3 (33.3)	8 (32.0)
Vomiting	1 (33.3)	3 (42.9)	1 (16.7)	5 (31.3)	1 (25.0)	1 (25.0)	1 (100.0)	3 (33.3)	8 (32.0)
Hypokalemia	2 (66.7)	2 (28.6)	2 (33.3)	6 (37.5)	0	1 (25.0)	0	1 (11.1)	7 (28.0)
Peripheral oedema	1 (33.3)	2 (28.6)	0	3 (18.8)	2 (50.0)	2 (50.0)	0	4 (44.4)	7 (28.0)
Weight decreased	1 (33.3)	2 (28.6)	1 (16.7)	4 (25.0)	1 (25.0)	2 (50.0)	0	3 (33.3)	7 (28.0)

**Table 3:** Differences in non-haematological adverse event incidence in 3-h and 24-h obatoclax infusion cohorts

	**3-h cohorts *n* (%)**	**24-h cohorts *n* (%)**
**⩾20% Difference in incidence of overall adverse events**		
*3-h > 24-h*	16 (100)	9 (100)
Somnolence	12 (75)	0 (0)
Alopecia	9 (56)	3 (33)
Constipation	9 (56)	3 (33)
Euphoric mood	7 (44)	1 (11)
Dysgeusia	6 (38)	0 (0)
Hypokalemia	6 (38)	1 (11)
Ataxia	5 (31)	1 (11)
Insomnia	5 (31)	0 (0)
Abdominal pain	4 (25)	0 (0)
Cough	4 (25)	0 (0)
Non-cardiac chest pain	4 (25)	0 (0)
		
*24-h > 3-h*		
Hyponatremia	1 (6)	4 (44)
Peripheral oedema	3 (19)	4 (44)
Asthenia	2 (13)	3 (33)
Deep vein thrombosis	1 (6)	3 (33)
Hyperglycemia	0 (0)	2 (22)
Neck pain	0 (0)	2 (22)
Pain	0 (0)	2 (22)
Wheezing	0 (0)	2 (22)
		
**⩾10% Difference in absolute incidence of grade 3 and 4 adverse events**		
*3-h > 24-h*	16 (100)	9 (100)
Somnolence	5 (31)	0 (0)
Hypokalemia	4 (25)	1 (11)
Diarrhoea	2 (13)	0 (0)
Nausea	2 (13)	0 (0)
		
*24-h > 3-h*		
Hyponatremia	0 (0)	4 (44)
Deep vein thrombosis	1 (6)	2 (22)
Chronic obstructive pulmonary disease	0 (0)	1 (11)
Dyspnea	0 (0)	1 (11)
INR increased	0 (0)	1 (11)
Neck pain	0 (0)	1 (11)
Pain	0 (0)	1 (11)
Pulmonary embolism	0 (0)	1 (11)

Abbreviation: INR = International Normalised Ratio.

**Table 4:** Grade 3 and 4 adverse events in two or more patients

	**3-h infusion schedule, *n* (%)**				**24-h infusion schedule, *n* (%)**				**Total, *n* (%)**
**System organ class preferred term**	**Obatoclax 15 mg per day (*n*=3)**	**Obatoclax 30 mg per day (*n*=7)**	**Obatoclax 45 mg per day (*n*=6)**	**Overall (*n*=16)**	**Obatoclax 30 mg per day (*n*=4)**	**Obatoclax 45 mg per day (*n*=4)**	**Obatoclax 60 mg per day (*n*=1)**	**Overall (*n*=9)**	**(*N*=25**)
*Neutropenia*									
Grade 3	0	1 (14.3)	1 (16.7)	2 (12.5)	0	1 (25.0)	0	1 (11.1)	3 (12.0)
Grade 4	3 (100.0)	4 (57.1)	3 (50.0)	10 (62.5)	4 (100.0)	2 (50.0)	1 (100.0)	7 (77.8)	17 (68.0)
									
*Thrombocytopenia*									
Grade 3	1 (33.3)	2 (28.6)	1 (16.7)	4 (25.0)	0	1 (25.0)	0	1 (11.1)	5 (20.0)
Grade 4	0	2 (28.6)	1 (16.7)	3 (18.8)	0	2 (50.0)	0	2 (22.2)	5 (20.0)
									
*Anaemia*									
Grade 3	0	2 (28.6)	1 (16.7)	3 (18.8)	0	2 (50.0)	0	2 (22.2)	5 (20.0)
Grade 4	0	0	1 (16.7)	1 (6.3)	0	0	0	0	1 (4.0)
									
*Hypokalemia*									
Grade 3	2 (66.7)	1 (14.3)	1 (16.7)	4 (25.0)	0	1 (25.0)	0	1 (11.1)	5 (20.0)
									
*Somnolence*									
Grade 3	0	3 (42.9)	2 (33.3)	5 (31.3)	0	0	0	0	5 (20.0)
									
*Fatigue*									
Grade 3	1 (33.3)	0	2 (33.3)	3 (18.8)	0	1 (25.0)	0	1 (11.1)	4 (16.0)
									
*Hyponatremia*									
Grade 3	0	0	0	0	1 (25.0)	2 (50.0)	0	3 (33.3)	3 (12.0)
Grade 4	0	0	0	0	1 (25.0)	0	0	1 (11.1)	1 (4.0)
									
*Deep vein thrombosis*									
Grade 3	0	1 (14.3)	0	1 (6.3)	0	2 (50.0)	0	2 (22.2)	3 (12.0)
									
*Leukopenia*									
Grade 3	0	1 (14.3)	1 (16.7)	2 (12.5)	0	0	0	0	2 (8.0)
Grade 4	0	1 (14.3)	0	1 (6.3)	0	0	0	0	1 (4.0)
									
*Diarrhoea*									
Grade 3	0	1 (14.3)	1 (16.7)	2 (12.5)	0	0	0	0	2 (8.0)
									
*Nausea*									
Grade 3	0	2 (28.6)	0	2 (12.5)	0	0	0	0	2 (8.0)

**Table 5:** Efficacy in intent-to-treat population

	**Complete or partial response, *n* (%)** a	**Median PFS, days (95% CI)**	**Median OS, days (95% CI)**
3-h infusion (*n*=16)	13 (81)^b^	184.5 (136–203)	379 (246–504)
24-h infusion (*n*=9)	4 (44)	181 (43–203)	283 (80–393)
All patients (*n*=25)	17 (68)	182 (158–198)	312 (246–393)

Abbreviations: CI=confidence interval; OS=overall survival; PFS=progression-free survival.

a All responses were confirmed by a second evaluation at least 4 weeks later.

b Includes two complete responses.
